# The Physiological Effects of Massage

**Published:** 1886-08

**Authors:** 


### *THE PHYSIOLOGICAL EFFECTS OF MASSAGE.*

Since in massage the physicians of to-day are placing some reliance in the treatment of various faults of nutrition, it is well to ascertain, so far as is possible, exactly its physiological effects. In the *Lancet* of May 22, 1886, appears an article, setting forth the results of massage on four medical students, who for three weeks were placed by Dr. F. Gopadze in Prof. Manassein's clinic, and lived only on bread, milk, veal, soup and roast beef; the quantities taken daily being accurately recorded. The nitrogen in the foods taken and in the excretions was determined by the Kjeldahl-Borodin process. Each was given massage from twenty to twenty-five minutes, once each day, two or three hours after eating. The appetites were increased and continued large after stopping the massage.

The nitrogenized transformations were increased in all the cases. Two gained in weight, while two lost, but the next week after massage ceased, they all gained flesh. After the massage, for about half an hour, the axillary temperature dropped from 0.10 to 0.5 C., after which it began to rise, and in an hour had nearly reached the normal. Respiration was increased in frequency, and deeper. The pulse varied with the character of the massage—slight massage made a more rapid pulse; more forcible manipulations produced a slower pulse.

